# Cerebrospinal fluid-contacting neurons: a promising source for adult neural stem cell transplantation in spinal cord injury treatment

**DOI:** 10.3389/fcell.2025.1549194

**Published:** 2025-03-12

**Authors:** Zhangrong Luo, Zeyu Shangguan, Liang Cao, Yi Zhang, Qizhe Li, Xuexing Shi, Jiangquan Fu, Chunqing Wang, Xiaowei Dou, Wei Tan, Qing Li

**Affiliations:** ^1^ Department of Emergency, The Affiliated Hospital of Guizhou Medical University, Guiyang, China; ^2^ Department of Traumatic Orthopedics, The Affiliated Yongchuan Hospital of Chongqing Medical University, Chongqing, China; ^3^ School of Clinical Medicine, Guizhou Medical University, Guiyang, China; ^4^ Clinical Research Center, Affiliated Hospital of Guizhou Medical University, Guiyang, China

**Keywords:** neural stem cells, cerebrospinal fluid-contacting neurons (CSF-cNs), spinal cord injury (SCI), cell transplantation therapy, motor function recovery

## Abstract

Transplantation of adult neural stem cells (NSCs) is regarded as one of the most promising approaches for treating spinal cord injury (SCI). However, securing a sufficient and reliable source of adult NSCs remains one of the primary challenges in applying this method for SCI treatment. Cerebrospinal fluid-contacting neurons (CSF-cNs) act as adult NSCs and can be substantially expanded *in vitro* while maintaining their NSC characteristics even after 60 passages. When CSF-cNs are transplanted into the injury sites of SCI mice, they demonstrate high survival rates along with the ability to proliferate and differentiate into neurons, astrocytes, and oligodendrocytes. Additionally, significant improvements in motor function have been observed in SCI mice following the transplantation of CSF-cNs. These results suggest that CSF-cNs may represent a promising source of adult NSCs for transplantation therapy in SCI.

## Introduction

Spinal cord injury (SCI) presents a formidable medical challenge leading to long-term disability, primarily due to the inherent deficiency in the central nervous system’s regenerative capacity, which hinders the effectiveness of traditional treatment methods in restoring post-injury function. Stem cell transplantation has emerged as a promising strategy to promote neural regeneration following SCI, with various stem cell types assessed for therapeutic potential in SCI treatment, such as embryonic stem cells (ESCs), induced pluripotent stem cells (iPSCs), adult neural stem/progenitor cells (NSCs/NPCs), and mesenchymal stem cells (MSCs) (Ceto et al., 2020; Rosenzweig et al., 2018; Sankavaram et al., 2019; Cao T. T. et al., 2022; Assinck et al., 2017). Adult NSCs are regarded as ideal candidates for cell transplantation due to their distinctive neural lineage differentiation capabilities, low tumorigenicity, and absence of ethical concerns (Fischer et al., 2020). Nevertheless, obtaining adult NSCs is one of the major technical challenges in using them to treat SCI. Initially, in 1992 and 1996, Reynolds and Weiss isolated NSCs from the adult mouse brain and spinal cord via a serum-free culture system for purification (Reynolds and Weiss, 1992; Gritti et al., 1996). Building on this, a team led by Lee and colleagues utilized fluorescence-activated cell sorting (FACS) to purify adult NSCs by selecting CD133 antigen-positive cells and eliminating neurons and oligodendrocytes through negative selection for polysialylated form of the neural cell adhesion molecule (PSA-NCAM) and O4 (Lee et al., 2005). Subsequently, various studies have continued to investigate different markers for isolating adult NSCs, such as EGFR, Lex, and glutamate-aspartate transporter (GLAST) (Tome-Garcia et al., 2017; Daniel Lacorazza, 2018). In conclusion, due to the absence of specific markers for the precise identification of adult NSCs (Chaker et al., 2016; Morales and Mira, 2019), adult NSCs obtained from mammalian tissue through these methods exhibit considerable heterogeneity (Pastrana et al., 2011; Deshpande et al., 2019; Siebzehnrubl et al., 2011). Therefore, establishing a reliable and effective source of these cells is crucial for the application of transplantation therapy in SCI utilizing adult NSCs.

Cerebrospinal Fluid-contacting Neurons (CSF-cNs) are unusual polymodal multifunctional cells situated at the interface between the cerebrospinal fluid and spinal cord parenchyma (Wyart et al., 2023; Vigh et al., 1977). CSF-cNs exhibit characteristics of immature neurons, which confers them with significant structural plasticity and the ability to respond and regenerate following SCI (Orts-Del'Immagine et al., 2014; Djenoune et al., 2017; Petracca et al., 2016; Orts-Del'Immagine et al., 2017). Our research has discovered that CSF-cNs in mice are activated and proliferate following SCI (Cao L. et al., 2022); CSF-cNs obtained via FACS are capable of forming neurospheres *in vitro* and differentiating into neurons and glial cells (Wang et al., 2021). These pieces of evidence indicates that CSF-cNs act as adult NSCs within the spinal cord. Nevertheless, it is still unclear whether CSF-cNs are suitable candidates for transplantation therapy in SCI.

In this study, we successfully engineered a lentivirus with a GFP reporter gene driven by the polycystic kidney disease 2-like 1 (Pkd2l1) promoter to isolate and label CSF-cNs derived from the cervical spinal cord of neonatal mice, which demonstrated excellent self-renewal capability *in vitro*. The transplanted CSF-cNs survived, proliferated, and differentiated *in vivo* in mice, indicating that CSF-cNs are suitable for transplantation therapy for SCI.

## Result

### Screening and characterization of CSF-cNs via Pkd2l1

**FIGURE 1:**
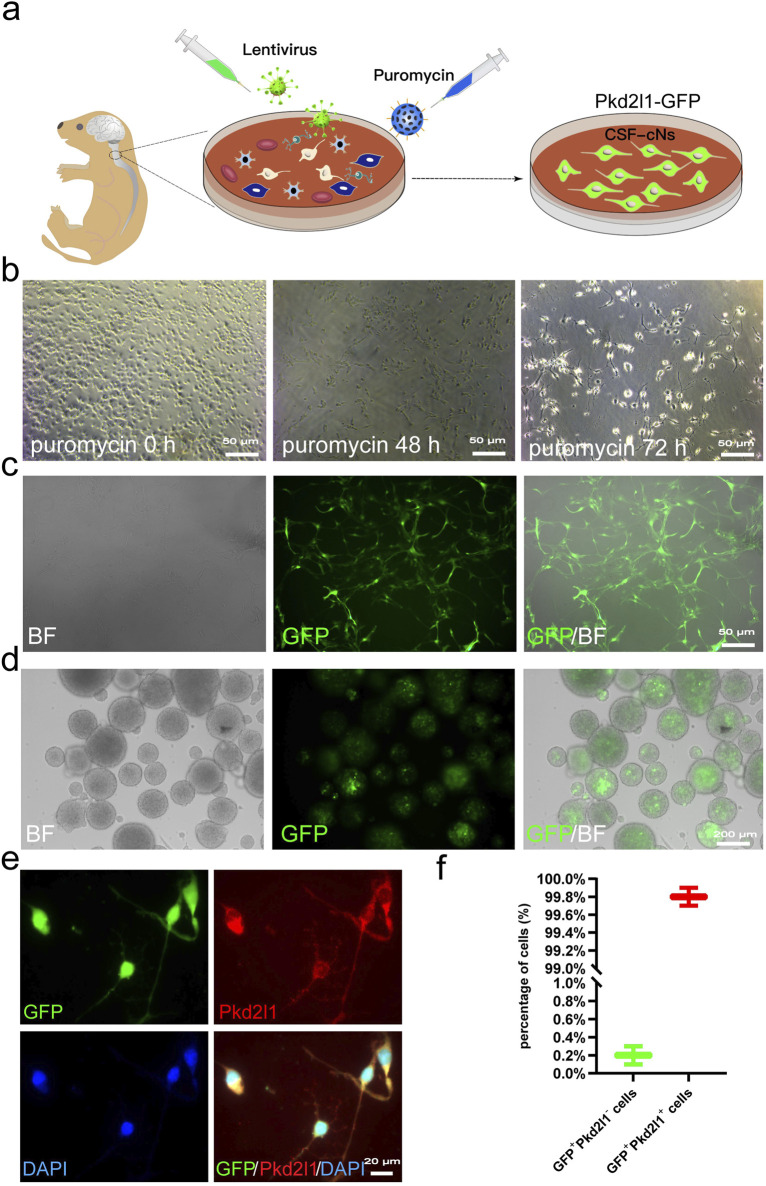
Screening and Labeling of CSF-cNs by Pkd2l1 **(A)** Screening process for high-purity CSF-cNs. Primary CSF-cNs were extracted from the cervical medullary regions of neonatal mice within 24 h of birth. Lentiral particles were to label CSF-cNs. Successfully labeled CSF-cNs screened using puromycin. The CSF-cNs expressed green fluorescent protein (GFP). **(B)** After transfecting primary cells containing CSF-cNs with lentivirus, puromycin (1 μg/mL) was added to select CSF-cNs. After 72 h, only a small number of adherent cells remained viable. **(C)** CSF-cNs that were successfully labeled by lentiviral particles could adhere to the wall and continue to grow. GFP expression was observed using inverted microscopy. BF = Bright Field. **(D)** Neurospheres formed by the suspension growth of CSF-cNs (GFP^+^ cells) in a single cell state. BF = Bright Field. **(E)** The results of cellular immunofluorescence showed that CSF-cNs (GFP^+^ cells) were co-labeled with their specific marker, Pkd2l1. **(F)** Quantitative analysis of CSF-cNs (GFP^+^ cells) co-labeled with Pkd2l1 revealed that nearly 100% (99.8%) of the GFP^+^ cells were indeed Pkd2l1^+^ CSF-cNs.

We constructed a lentiviral reporter gene driven by the Pkd2l1 promoter, which has been confirmed as a specific marker for CSF-cNs (Orts-Del'Immagine et al., 2014; Huang et al., 2006; Djenoune et al., 2014), in order to screen CSF-cNs *in vitro* (Figure 1A). From the cervical spinal cord tissue of neonatal C57BL/6 mice with 24 h after birth, we isolated a cell mixture containing primary CSF-cNs. By employing lentiviral transduction and subsequent puromycin selection from the cell mixture, CSF-cNs were effectively isolated (Figure 1B). Cells that survived successful transduction expressed the GFP reporter gene (Figure 1C). These GFP^+^ cells were then cultured in suspension and supplemented with the growth factors Epidermal Growth Factor (EGF) and basic Fibroblast Growth Factor (bFGF) to enhance their expansion. After 3 days, GFP^+^ cells formed neurospheres (Figure 1D). The neurospheres were subsequently dissociated into single cells and subjected to Pkd2l1 immunofluorescence staining, exhibiting that nearly all cells were co-labeled with GFP and Pkd2l1 (Figures 1E,F, with 99.8% co-labeling).

### Expansion of CSF-cNs *in vitro*

**FIGURE 2:**
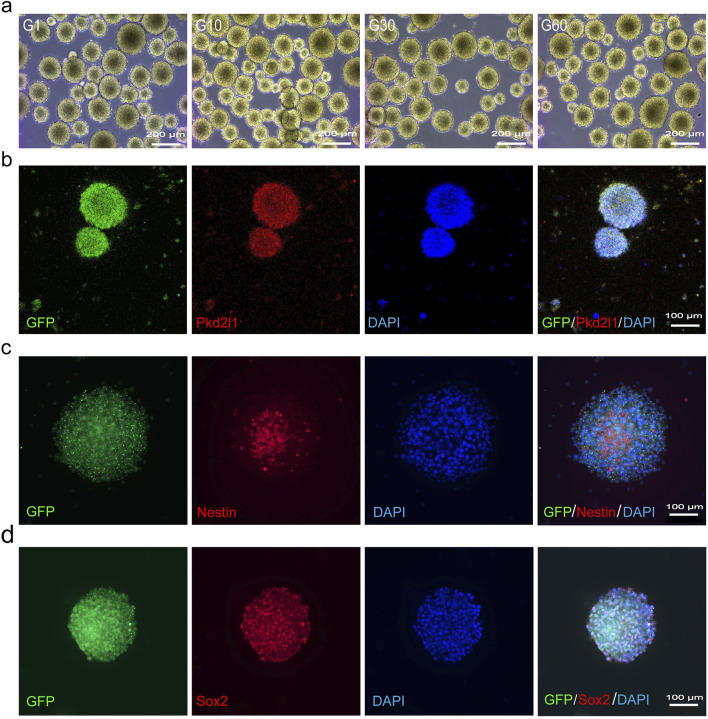
CSF-cNs can be continuously passaged *in vitro* **(A)** Bright-field images of CSF-cNs suspension culture *in vitro* under an inverted microscope. CSF-cNs possess an excellent spheroid-forming ability *in vitro*. CSF-cNs in the unicellular state can form a large number of neurospheres with a diameter of 150–200 μm in 3–4 days and can be continuously passaged up to a maximum of 60 generations *in vitro*. **(B)** Cellular immunofluorescence images showing neurospheres formed by CSF-cNs (GFP^+^ cells) co-labeled with their specific marker Pkd2l1. **(C, D)** Cellular immunofluorescence images showing that neurospheres formed by CSF-cNs (GFP^+^ cells) expressed the neural stem cell markers Nestin, Sox2. Nestin is expressed in the cell membrane, Sox2 is expressed in the nucleus. Nuclei are counterstained with DAPI in all panels.

**FIGURE 3:**
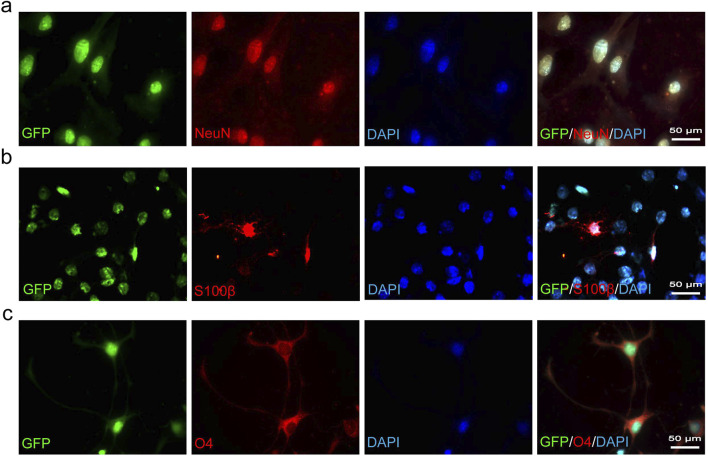
CSF-cNs demonstrate trilineage differentiation potential *in vitro*. **(A–C)** Representative immunofluorescence images of CSF-cNs following induced differentiation. The GFP^+^ CSF-cNs were subjected to differentiation induction for 7 days *in vitro*. These GFP^+^ cells were immunofluorescently co-labeled with the neuronal marker NeuN **(A)**, the astrocyte marker S100β **(B),** and the oligodendrocyte marker O4 **(C)**. Cell nuclei in all images were counterstained with DAPI.

To acquire sufficient cells for transplantation therapy, adult NSCs typically require extensive *in vitro* expansion before use. Following the introduction of growth factors EGF and bFGF into the suspension culture of CSF-cNs, we observed the formation of neurospheres, which could be passaged after 3–4 days of cultivation (Figure 2A). Immunofluorescence staining of neurospheres formed from the 60th generation of CSF-cNs revealed the expression of Pkd2l1, Nestin, and Sox2 (Figures 2B–D). These findings indicate that CSF-cNs can be effectively passaged while retaining stem cell characteristics. To further validate the differentiation potential of CSF-cNs, the neurospheres were dissociated into single cells and subjected to adherence differentiation culture with 1% fetal bovine serum (FBS). The results showed that CSF-cNs retain the capacity to differentiate into neurons, astrocytes, and oligodendrocytes *in vitro* (Figures 3A–C).

### The transplanted CSF-cNs can survive and express NSC markers in SCI mice

**FIGURE 4:**
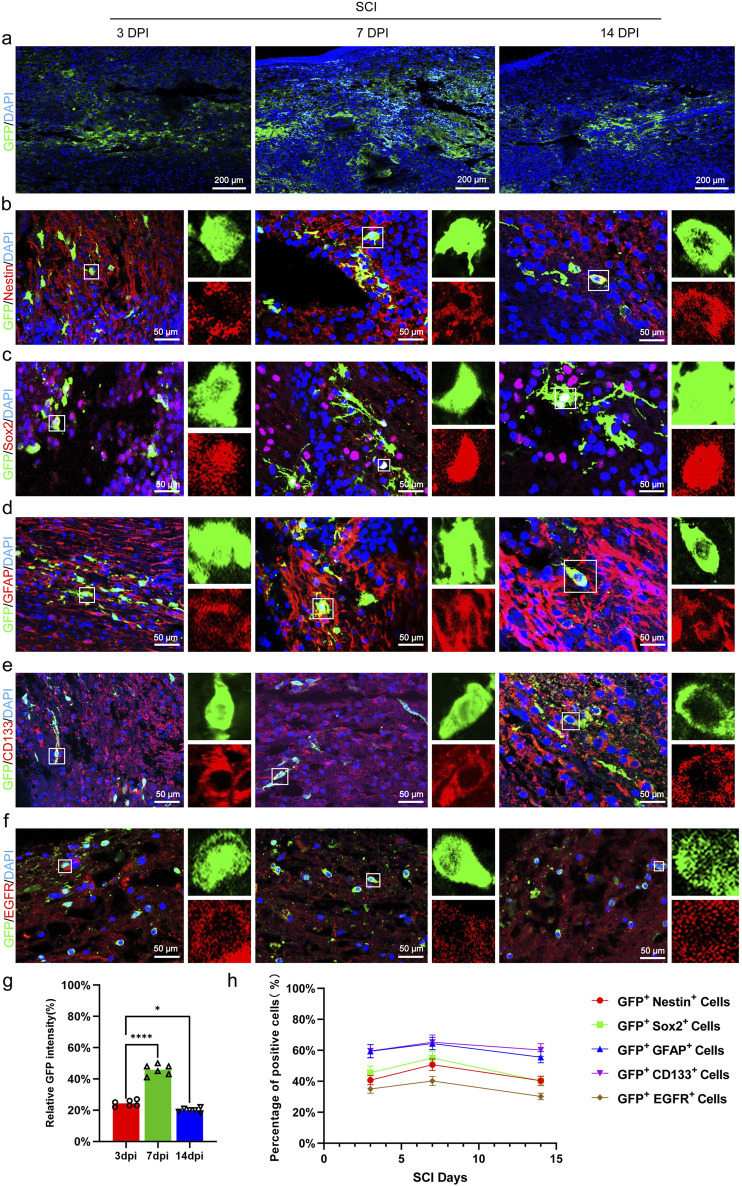
Transplanted CSF-cNs exhibit characteristics of adult NSC. **(A)** Representative images of CSF-cN grafts at 3, 7, and 14 days post-transplantation. Immunohistochemical staining (IHC) detected CSF-cNs (GFP + cells) at these time points, indicating the survival of transplanted CSF-cNs (GFP^+^ cells) in spinal cord-injured mice. **(B–F)** Representative IHC images of CSF-cNs (GFP^+^ cells) transplanted into spinal cord-injured mice at 3, 7, and 14 days. White boxes indicate co-labeled positive cells, with the enlarged sections on the right showing the positive cells within the boxes. At 3, 7, and 14 days post-transplantation, CSF-cNs (GFP^+^ cells) expressed neural stem cell markers Nestin **(B)**, Sox2 **(C),** GFAP **(D)**, CD133 **(E)**, and EGFR **(F)**. Among these markers, EGFR **(F)** indicates the activated state, while GFAP **(D)** and CD133 **(E)** indicate the quiescent state of neural stem cells.**(G, H)** Quantitative analysis of co-labeled cells after transplantation of CSF-cNs (GFP^+^ cells) into spinal cord-injured mice **(A–F)**. DPI = days post-injury, SCI = spinal cord injury, N = 6.

We utilized immunofluorescence to observe GFP^+^ cells in order to evaluate the survival of transplanted CSF-cNs in mice. The results showed that CSF-cNs survived at 3 days post-transplantation, increased to a peak at 7 days, and then declined by 14 days, yet still maintained a high count similar to that observed on day 3 (Figures 4A,G). We further observed that the transplanted CSF-cNs expressed NSC markers, Nestin and Sox2 (Figures 4B,C). Among these, a subset of cells expressed GFAP and CD133 (quiescent stem cell markers, Figures 4D,E), while others expressed EGFR (activation stem cell marker, Figure 4F) (Codega et al., 2014; Dulken et al., 2017).

### The transplanted CSF-cNs possess the ability to proliferate and differentiate *in vivo*

**FIGURE 5:**
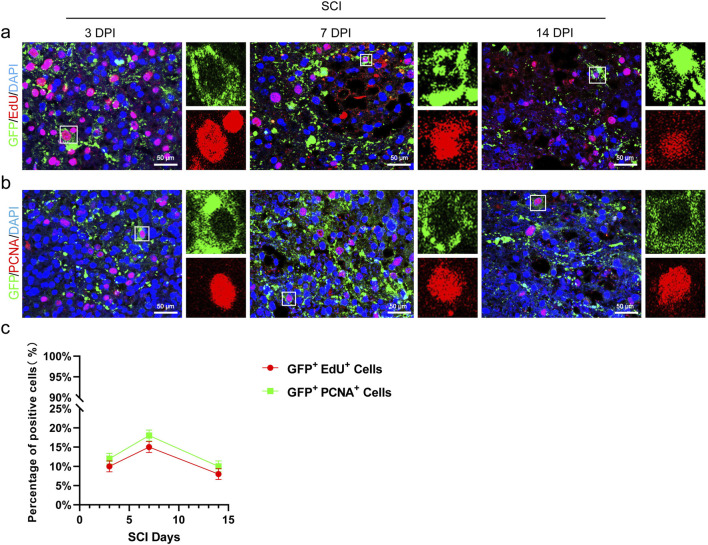
Transplanted CSF-cNs possess proliferative capacity. **(A)** Representative images from the EdU incorporation assay show the proliferation of CSF-cNs (GFP^+^ cells) at 3, 7, and 14 days after transplantation into spinal cord-injured mice. The GFP + CSF-cNs were observed to proliferate at various time points post-transplantation. Enlarged sections on the right highlight co-labeled positive cells within the white boxes. **(B)** Representative IHC images display the proliferation of CSF-cNs (GFP^+^ cells) at 3, 7, and 14 days following transplantation into spinal cord-injured mice. At these time points, CSF-cNs (GFP^+^ cells) expressing the proliferation marker PCNA were detected via IHC. Enlarged sections on the right show co-labeled positive cells within the white boxes. **(C)** Quantitative analysis of the results from experiments **(A, B)**. The data indicate that the proportion of proliferating CSF-cNs (GFP^+^ cells) peaked at day 7 post-transplantation. DPI = days post-injury, SCI = spinal cord injury, N = 6.

We utilized EdU incorporation assays to examine the proliferation of transplanted CSF-cNs in mice. The results showed that EdU^+^/GFP^+^ cells were observed on the third, 7th, and 14th days post-transplantation, peaking on the 7th day and declining by the 14th day (Figures 5A–C). Further immunofluorescence analysis of the proliferation marker PCNA in transplanted CSF-cNs demonstrated results consistent with those of the EdU incorporation assay (Figures 5B,C).

**FIGURE 6:**
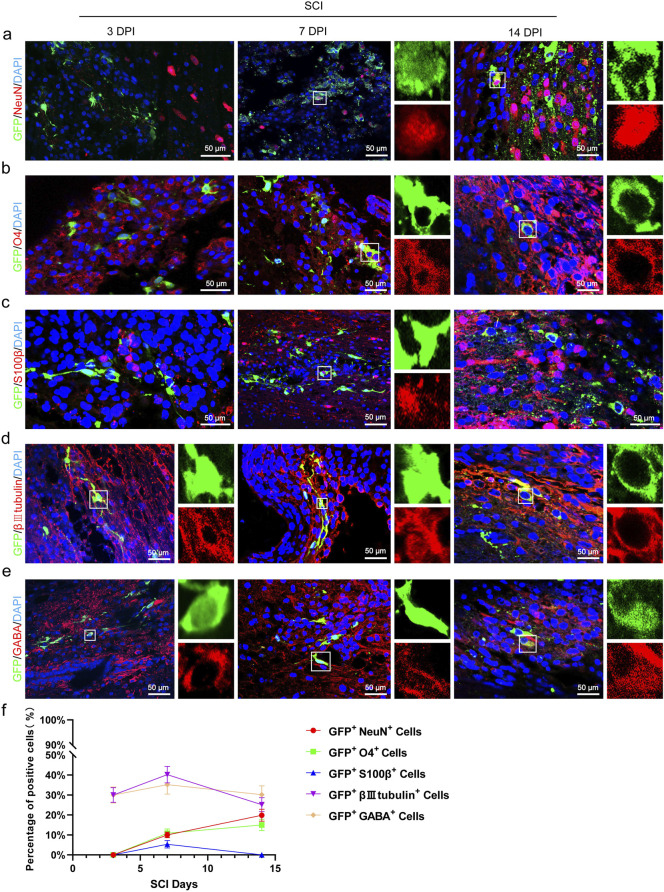
Transplanted CSF-CNs exhibit multipotential differentiation ability *in vivo*. **(A)** Representative images showing CSF-cNs (GFP^+^ cells) co-labeled with the mature neuron marker NeuN. Co-labeling with NeuN was observed in CSF-cNs (GFP^+^ cells) at 7 and 14 days post-transplantation. **(B)** Representative images showing CSF-cNs (GFP^+^ cells) co-labeled with the oligodendrocyte marker O4. Co-labeling with O4 was detected in CSF-cNs (GFP^+^ cells) at 7 and 14 days post-transplantation. **(C)** Representative images showing CSF-cNs (GFP^+^ cells) co-labeled with the astrocyte marker S100β. Co-labeling with S100β was observed in CSF-cNs (GFP^+^ cells) at 7 days post-transplantation. **(D)** Representative images showing transplanted CSF-cNs (GFP^+^ cells) co-labeled with the immature neuron marker βIII tubulin. Co-labeling with βIII tubulin was detected in CSF-cNs (GFP^+^ cells) at 3, 7, and 14 days post-transplantation. **(E)** Representative images showing CSF-cNs (GFP^+^ cells) co-labeled with the GABA marker. Co-labeling with GABA was detected in CSF-cNs (GFP^+^ cells) at 3, 7, and 14 days post-transplantation. White boxes indicate co-labeled positive cells; the enlarged sections on the right side of images **(A–E)**(a–e) show the positive cells within the boxes. **(F)** Quantitative analysis of the results from experiments **(A–E)**. By day 7 post-transplantation, CSF-cNs (GFP^+^ cells) began differentiating into mature neurons and oligodendrocytes, with further increases observed by day 14. Conversely, differentiation into a small number of astrocytes was detected only at day 7 post-transplantation and had disappeared by day 14. DPI = days post-injury, SCI = spinal cord injury, N = 6.

To verify whether transplanted CSF-cNs have the capacity of multipotent differentiation *in vivo*, We performed immunofluorescence staining on transplanted CSF-cNs using NeuN (a mature neuronal marker), S100β (an astrocyte marker), and O4 (an oligodendrocyte marker). The results showed that on 3 days post-transplantation, NeuN^+^/GFP^+^, S100β^+^/GFP^+^, and O4^+^/GFP^+^ cells were not detected. However, by 7 days post-transplantation, all these types of cells were observed. At 14 days post-transplantation, NeuN^+^/GFP^+^ and O4^+^/GFP^+^ double-positive cells further increased, whereas S100β^+^/GFP^+^ double-positive cells were not observed (Figures 6A–C,F). Notably, the transplanted CSF-cNs expressed the immature neuronal marker βIII-tubulin and the GABAergic neuron marker GABA at 3-, 7-, and 14-days post-transplantation, indicating that post-transplantation CSF-cNs possess characteristics of immature neurons and GABAergic neurons (Figures 6D–F).

### CSF-cNs transplantation improves hindlimb motor function after SCI

**FIGURE 7:**
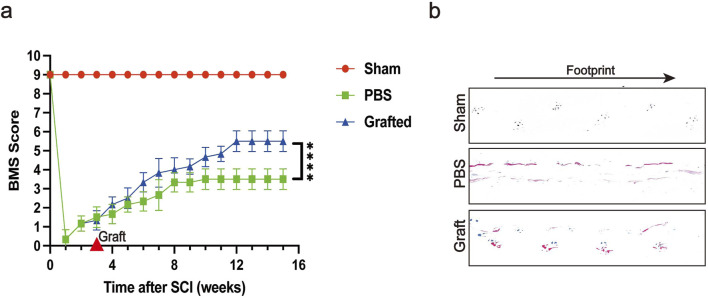
Transplanted CSF-cNs ameliorate hindlimb motor function of SCI mice. **(A)** The trend of Basso Mouse Scale (BMS) scores at various time points post-SCI is shown for the Sham group, SCI group, and CSF-cNs transplantation group. Mice in the CSF-cNs transplantation group exhibited higher BMS scores than those in the SCI group. Note: CSF-cNs transplantation was performed in the third week after SCI in mice. Two-way ANOVA followed by Šídák multiple comparison test, ****P < 0.0001. N = 6. **(B)** Eight weeks post-transplantation, footprint analysis revealed that the footprints of mice in the CSF-cNs transplantation group were more similar to those of the Sham group compared to the SCI group.

Contusion injury is the most frequently employed model in experimental research, as the majority of SCI patients experience this type of trauma (Guan et al., 2023). We used a forceps contusion method to induce SCI in mice. The forceps method results in an SCI model where it is challenging to ensure consistent severity of neural injury; therefore, 3 weeks post-SCI, we selected mice with uniform levels of paralysis for transplantation. The Basso Mouse Scale for Locomotion (BMS) is a scoring system used to evaluate motor function in mice, and we conducted BMS assessments on SCI mice following the transplantation of CSF-cNs (Figure 7A). The results showed that the enhancement of BMS scores in the transplantation group was significantly greater than that in the control group. We further evaluated hindlimb coordination and stability in post-SCI mice via footprint analysis (Metz and Whishaw, 2002). By the 12th week post-transplantation of CSF-cNs, we observed that compared to control mice, which exhibited hindlimb dragging during movement, the transplanted mice showed improved symmetry and uniformity in stride length and width (Figure 7B). In conclusion, BMS scoring and footprint analysis demonstrated that CSF-cNs transplantation effectively promote the recovery of motor function in mice following SCI (Pajoohesh-Ganji et al., 2010).

## Discussion

In this study, we examined the potential of CSF-cNs, which act as NSCs, in the context of transplantation therapy for SCI, with a particular emphasis on their self-renewal ability *in vitro*. In fact, expansion of adult NSCs still faces numerous challenges *in vitro*, particularly regarding the large-scale production needed for clinical applications. Due to the heterogeneity of adult NSCs within the spinal cord (Chaker et al., 2016; Bond et al., 2015; Obernier and Alvarez-Buylla, 2019), significant variability exists in the stem cell potential of currently obtained adult NSCs *in vitro*. Neurospheres previously derived from the adult human spinal cord lacked the capacity for long-term passaging, thereby hindering large-scale production and impeding future therapeutic applications (Akesson et al., 2007; Dromard et al., 2008). Moreover, adult NSCs derived from the human spinal cord undergo genetic alterations during long-term expansion *in vitro* (Mothe and Tator, 2015). Our results demonstrate that CSF-cNs can be extensively expanded under serum-free culture conditions while maintaining stable NSC characteristics during passaging. The robust self-renewal capacity of CSF-cNs *in vitro* highlights their superior potential for application in SCI transplantation therapy.

The survival of transplanted cells is crucial for their efficacy. Immunorejection is a major challenge affecting the survival rate of transplanted stem cells. Many studies necessitate the use of immunosuppressants both prior to and following cell transplantation or conduct transplantation experiments in immunodeficient animal models to enhance cell survival (Karimi-Abdolrezaee et al., 2010; Hawryluk et al., 2014; Lu et al., 2012; Salazar et al., 2010). Previous studies have indicated that adult NSCs possess lower immunogenicity (Mammolenti et al., 2004; Liu et al., 2021). In our study, we directly transplanted CSF-cNs into the spinal cord of wild-type SCI mice without using immunosuppressants. We observed that the transplanted CSF-cNs could survive in the SCI mice. This evidence further suggests that CSF-cNs may be a low-immunogenic adult NSC in the mouse spinal cord. In addition, the timing of transplantation is a critical factor in ensuring post-transplantation cell survival. The early stages of SCI produce many neurotoxins, creating a hostile environment that is unfavorable for the survival of transplanted stem cells (Rouanet et al., 2017; Medalha et al., 2014). Consequently, more research opts for transplantation during the subacute phase of SCI to enhance the survival rate of transplanted cells (Shang et al., 2022). However, cell transplantation during the acute phase of SCI can effectively mitigate secondary damage and more efficiently promote neurological recovery (Watanabe et al., 2015). We performed *in situ* transplantation immediately after SCI in mice and observed that CSF-cNs transplanted during the acute phase of SCI maintained an elevated survival rate 14 days post-transplantation, with negligible difference from the survival rate observed on day 3. These discoveries indicate that CSF-cNs possess strong resilience in the spinal cord of SCI mice.

The ideal goal of stem cell transplantation in the treatment of spinal cord injury is for the transplanted stem cells to differentiate and replace lost cells. Previous evaluations have highlighted the potential of adult NSCs from various sources in transplantation therapy for SCI, mediating functional improvements post-SCI through multiple mechanisms including cell replacement, neuroprotection, immunomodulation, promoting remyelination, and enhancing axonal regeneration and sprouting (Hawryluk et al., 2012; De Gioia et al., 2020; Cao et al., 2010). In fact, without external intervention, the differentiation trajectory of adult NSCs transplanted *in vivo* is not arbitrary, with a preference toward neuronal differentiation being rare. The transplanted adult NSCs typically differentiate into glial cells, particularly oligodendrocytes (Sankavaram et al., 2019; Karimi-Abdolrezaee et al., 2010). In contrast, our observations suggest that transplanted CSF-cNs undergo *in vivo* differentiation, primarily towards neuronal and oligodendrocyte lineages, and can differentiate into GABA neurons. This may be a crucial factor in the CSF-cNs transplantation promoting the recovery of motor function in the SCI mice. However, this study did not explore deeply the specific mechanisms of action.

In summary, our research is the first to reveal the potential of CSF-cNs as adult NSCs in transplantation therapy following SCI. The focus of future research will concentrate on further elucidating the mechanisms by which transplanted CSF-cNs promote functional recovery after spinal cord injury. Accomplishing this work will pave the way for using CSF-cNs in transplantation therapies for SCI.

## Methods

### Animals

We acquired 6–8-week-old C57BL/6 mice from the Experimental Animal Center at Guizhou Medical University (License No.: SCXK [Guizhou] 2018–0001). All animal experimental procedures were approved by the Animal Care and Use Committee of Guizhou Medical University. During the study, the animals had unrestricted access to food and water and were housed in groups in standard polycarbonate cages under a 12-h light/dark cycle (lights on from 6:00 a.m. to 6:00 p.m.). The environmental temperature of the animal facility was maintained at 20°C–23°C with a relative humidity of 30%–70%.

### Isolation and culture of CSF-cNs

C57BL/6 mice for 24 h old were placed on ice for hypothermic anesthesia, followed by immersion in 75% alcohol for 5 min inside a biosafety cabinet in an SPF-grade animal room, and then euthanized via decapitation. The cervical spinal cord tissue was isolated on ice and transferred to dissection medium (DMEM-HG medium +5% penicillin-streptomycin +20 ng/mL bFGF +20 ng/mL EGF). Using sterile ophthalmic scissors, the target tissue was repeatedly and evenly minced into small pieces approximately 0.5 mm^3^. The tissue fragments were transferred to a papain solution and digested at 37°C for 30 min, followed by centrifugation at 200 g for 5 min. The supernatant was discarded, and the pellet was resuspended in fresh serum-free neural stem cell medium (Neurobasal-A medium +2% B27 + 1% penicillin +1% L-glutamine +20 ng/mL bFGF +20 ng/mL EGF), then transferred to a 24-well plate pre-coated with 0.1 mg/mL poly-D-lysine (PDL) for adherent culture.

### Selection and purification of CSF-cNs

Primary cells were allowed to adhere for 1 h, after which lentivirus (Lentivirus-Pkd2l1-GFP-puromycin, Shanghai Genechem Co., Ltd.) was added to the medium for transfection over 24 h. Subsequently, the medium was replaced with fresh serum-free neural stem cell medium, and puromycin (2 g/mL, 3 μL) was added to select cells for 72 h. The surviving cells after selection were collected, adjusted to a cell density of 2 × 10^5/well, resuspended in fresh serum-free neural stem cell medium, and seeded into ultra-low attachment 6-well plates (Corning Inc., United States of America) for suspension culture in an incubator at 37°C with 5% CO2.

### Passaging of CSF-cNs

During the cultivation of CSF-cNs to form neurospheres, the entire culture medium was replaced every 3 days, facilitating the formation of neurospheres with diameters ranging from approximately 150–200 μm within 3–4 days. Once the neurospheres reached this size, they were carefully transferred to a centrifuge tube and centrifuged at 100 g for 5min, after which the supernatant was discarded. Accutase was added for 30 min to digest the cells, which were then mechanically dissociated into single cells. The cells were centrifuged again at 200×g for 5 min, with the supernatant discarded. They were then resuspended in fresh serum-free neural stem cell medium and continued with the same culture process as before.

### Induced differentiation of CSF-cNs

Single cells from the 61st passage of CSF-cNs were cultured for adhesion on pre-treated PDL-coated culture slides (Corning Inc.). The serum-free neural medium was replaced with a serum-containing differentiation medium (Neurobasal-A medium +1% penicillin +1% L-glutamine +1% fetal bovine serum), and after 7 days of culture, the differentiation ability of CSF-cNs was assessed via immunofluorescence.

### Spinal cord injury

SCI was induced at the T10 thoracic level in mice. All surgeries were performed under deep anesthesia, using a combination of ketamine (25 mg/kg), xylazine hydrochloride (5.8 mg/kg), chlorpromazine (0.25 mg/kg), and inhalation of isoflurane (0.5%–1%). Following a laminectomy at T10, a forceps with a diameter of 1.5 mm (McHugh Milieux, Downers Grove, IL) was inserted 0.8 mm below the surface of the exposed spinal cord and compressed for 10 s to induce SCI (O'Shea et al., 2022). Post-surgery, animals were returned to their respective cages and manually voided twice daily until reflexive bladder control was regained.

### *In vivo* transplantation of CSF-cNs

After establishing the spinal cord injury (SCI) model in mice, CSF-cNs transplantation was performed at two distinct time points. The first transplantation was conducted immediately after the induction of the SCI model, aiming to investigate the *in vivo* survival capacity of CSF-cNs during the acute phase of spinal cord injury (as shown in Figures 4–6). The second transplantation was performed 3 weeks after the establishment of the SCI model, selecting spinal cord-injured mice with identical BMS scores (as shown in Figure 7). The rationale for choosing this time point is that transplantation 3 weeks post-injury more closely mirrors the timing of clinical transplantation therapy in spinal cord injury patients. Our goal was to simulate real clinical scenarios as accurately as possible to evaluate the therapeutic efficacy of CSF-cNs transplantation. The neurospheres from the 60th passage of CSF-cNs, at their optimal growth state, were dissociated into single cells using Accutase. The cells were then concentrated in PBS to a density of 1.0 × 10^6^/mL for transplantation. Following the full exposure of the spinal cord tissue at the injury site in mice, 2 μL of the CSF-cNs single-cell suspension were gradually injected into both ends of the SCI lesion site using a microinjector. After the injection, the needle was left in place for 2 min, and the puncture site was sealed with medical adhesive to reduce the risk of cell suspension leakage. After successful cell transplantation, the muscle and skin were meticulously sutured in layers. The wound was then thoroughly disinfected, and lost fluids were replenished post-operatively with an intraperitoneal injection of saline. Upon completion of the surgery, all mice in the SCI model group were routinely administered 40,000 units of penicillin sodium to prevent infection (Lu et al., 2022). Post-surgery, manual urination was performed twice daily until reflexive bladder control was re-established.

### Immunocytochemistry

After removing the cell supernatant, the cells were washed three times with PBS, each for 5 min. The cells were then fixed at room temperature with 4% paraformaldehyde (PFA) for 15 min, followed by another three washes with PBS, each for 5 min. The cells were blocked at room temperature with a blocking solution composed of 10% goat serum and 0.3% Triton X-100 in PBS for 1 h. After removing the blocking solution, primary antibodies were added. The primary antibodies used in this study for cell immunofluorescence included Pkd2l1 (AB9084, 1:700, Merck Millipore, MA, United States), Nestin (sc23927, 1:200, Santa Cruz Biotechnology, Dallas, TX, United States), Sox2 (1:500, CST, Danvers, MA, United States), NeuN (MAB377, 1:400, Millipore, United States), S100β (66616-1-lg, 1:500, Proteintech, United States), and O4 (MAB1326, 1:400, RD, United States). The specimens were incubated overnight at 4°C. The following day, the specimens were warmed to room temperature for 60 min, the primary antibodies were removed, and the specimens were washed three times with PBS, each for 5 min. Goat anti-rabbit Alexa Fluor 488 (1:500, CST, Danvers, MA, United States) or goat anti-mouse Alexa Fluor 555 (1:500, CST, Danvers, MA, United States) was then added, and the cells were incubated at 37°C in the dark for 2 h. After the secondary antibodies were removed, the cells were washed five times with PBS, each for 5 min. The nuclei were stained with 4′,6-diamidino-2-phenylindole dihydrochloride (DAPI; 2 ng/mL; Molecular Probes) for 15 min and then observed under an inverted fluorescence microscope (Carl Zeiss AG, Germany).

### Immunohistochemistry

After anesthetizing the mice, cardiac perfusion was performed sequentially with 0.9% saline and 4% paraformaldehyde solution. Spinal cord tissue (2.0 cm) was collected centering on the lesion site and sectioned into spinal cord slices (5 μm thick) using a microtome. The tissue was fixed in 4% paraformaldehyde for 24 h and then embedded in paraffin. After deparaffinization, the tissue was permeabilized using a solution containing 0.25% Triton X-100, followed by blocking with a goat serum solution for 1 h. The primary antibodies used in this study included Pkd2l1 (AB9084, 1:700, Merck Millipore, MA, United States), Nestin (sc23927, 1:200, Santa Cruz Biotechnology, Dallas, TX, United States), Sox2 (1:400, CST, Danvers, MA, United States), GFAP (1:300, CST, Danvers, MA, United States), CD133 (66666-1-lg, 1:400, Proteintech, United States), EGFR (66455-1-lg, 1:500, Proteintech, United States), PCNA (10205-2-AP, 1:300, Proteintech, United States), NeuN (MAB377, 1:400, Millipore, United States), O4 (MAB1326, 1:400, RD, United States), β III tubulin (66240-1-Ig, 1:200, Proteintech, United States), S100β (66616-1-lg, 1:500, Proteintech, United States), and GABA (A2052, 1:200, Millipore, United States). The sections were incubated with the primary antibodies overnight at 4°C. Following three washes with PBS, the sections were incubated in the dark with goat anti-rabbit Alexa Fluor 488 (1:500, CST, Danvers, MA, United States) or goat anti-mouse Alexa Fluor 555 (1:500, CST, Danvers, MA, United States) for 1 h. Nuclei were stained using 4′,6-diamidino-2-phenylindole dihydrochloride (DAPI; 2 ng/mL; Molecular Probes). Anti-fade reagent (InVitrogen, Grand Island, NY) was applied for mounting. Results were recorded using a Leica confocal microscope (Carl Zeiss AG, Germany).

### Staining and imaging of 5-ethynyl-2′-deoxyuridine (EdU)

The specific details have been previously described in-depth (Cao L. et al., 2022). A dose of 50 mg/kg of 5-ethynyl-2′-deoxyuridine (EdU, Riobio, Guangzhou, China) was administered to mice via intraperitoneal injection. The tissue processing was similar to the immunofluorescence staining procedure. Following immunofluorescence staining, sections were stained using the Cell-light EdU Apollo567 and EdU kit (Riobio, Guangzhou, China), following the manufacturer’s instructions. The results were observed using a Leica confocal microscope.

### Image analysis

All image analyses were conducted by at least two independent researchers using ImageJ software. Initially, the region of interest (ROI) around the transplantation boundaries was delineated for each individual image using GFP immunoreactivity images. Automated cell counting methods were consistently verified against manual counts. Samples with poor immunostaining were excluded from the analysis. Any samples lacking slices with discernible transplanted tissue were also excluded. Generally, for a given sample, a series of 1 in 6 sections contained 3-5 slices with GFP signals. For tissue sections from animals with only lesions, the ROI was drawn along the lesion boundaries (defined by GFP immunoreactivity) and extended 500 μm outward to encompass both the lesion and its surrounding area for quantitative analysis.

### Statistical analysis

Statistical analysis was conducted using GraphPad Prism 9 (GraphPad Software, Inc.; La Jolla, CA). Data are presented as mean ± standard deviation and were analyzed using two-way ANOVA followed by Šídák multiple comparison test.A *P*-value of <0.05 was considered statistically significant.

## Data Availability

The original contributions presented in the study are included in the article/supplementary material, further inquiries can be directed to the corresponding authors.
